# Effects of Liposomes Contained in Thermosensitive Hydrogels as Biomaterials Useful in Neural Tissue Engineering

**DOI:** 10.3390/ma10101122

**Published:** 2017-09-22

**Authors:** Yusser Olguín, Cristian Campos, Javiera Catalán, Luís Velásquez, Fernando Osorio, Iván Montenegro, Alejandro Madrid, Cristian Acevedo

**Affiliations:** 1 Center for Integrative Medicine and Innovative Science (CIMIS), Facultad de Medicina, Universidad Andrés Bello, Echaurren 183, Santiago 8320000, Chile; luis.velasquez@unab.cl; 2 Departamento de Química, Facultad de Ciencias, Universidad de Chile, Las Palmeras 3425 Ñuñoa, Santiago 8320000, Chile; cristian.ciencias@gmail.com; 3 Escuela de Ingeniería en Biotecnología, Facultad de Ciencias Biológicas, Universidad Andres Bello, República 239, Santiago 8320000, Chile; j.catalanrozas93@gmail.com; 4 Department of Food Science and Technology, Universidad de Santiago de Chile, Avenida Ecuador 3769, Santiago 8320000, Chile; fernando.osorio@usach.cl; 5 Escuela de Obstetricia y Puericultura, Facultad de Medicina, Campus de la Salud, Universidad de Valparaíso, Angamos 655, Reñaca, Viña del Mar 2520000, Chile; ivan.montenegro@uv.cl; 6 Departamento de Química, Facultad de Ciencias Naturales y Exactas, Universidad de Playa Ancha, Avda, Leopoldo Carvallo 270, Playa Ancha, Valparaíso 2340000, Chile; alejandro.madrid@upla.cl; 7 Centro de Biotecnología, Universidad Técnica Federico Santa María, Avenida España 1680, Valparaíso 2340000, Chile; cristian.acevedo@usm.cl; 8 Departamento de Física, Universidad Técnica Federico Santa María, Avenida España 1680, Valparaíso 2340000, Chile; 9 Centro Científico Tecnológico de Valparaíso, Universidad Técnica Federico Santa María, Avenida España 1680, Valparaíso 2340000, Chile

**Keywords:** Pluronic F127/Chitosan hydrogels, growth of neurites PC12 cells, effect of Liposomes

## Abstract

Advances in the generation of suitable thermosensitive hydrogels for the delivery of cells in neural tissue engineering demonstrate a delicate relationship between physical properties and capabilities to promote cell proliferation and differentiation. To improve the properties of these materials, it is possible to add liposomes for the controlled release of bioactive elements, which in turn can affect the physical and biological properties of the hydrogels. In the present investigation, different hydrogels based on Pluronic F127 have been formulated with the incorporation of chitosan and two types of liposomes of two different sizes. The rheological and thermal properties and their relation with the neurite proliferation and growth of the PC12 cell line were evaluated. Our results show that the incorporation of liposomes modifies the properties of the hydrogels dependent on the concentration of chitosan and the lipid type in the liposomes, which directly affect the capabilities of the hydrogels to promote the viability and differentiation of PC12 cells.

## 1. Introduction

The scaffold formulation in neural tissue engineering provides adequate conditions for the growth of the transplanted cells. It is a strategy widely studied for the generation of therapeutic options in different neurological lesions through the filling of the cystic cavity, the guided promotion of cell migration and the development of neurite outgrowth. In this sense, several materials have been studied for use in neural tissue engineering [1,2]. However, one of the most used for its physical characteristics is hydrogels, which emulate the native characteristics of the extracellular matrix of the nervous system. Therefore, there is great interest in obtaining new formulations that provide the necessary evidence for the use of hydrogels in neural therapy [3,4,5,6,7,8,9]. In this context, thermosensitive hydrogels represent an adequate alternative due to their biocompatibility, biodegradation and formulation as injectable fluids that can form a gel in the organism before solidifying within the desired tissue, where Pluronic F127 stands out as a possible therapeutic alternative [10,11,12].

Pluronic F127 corresponds to poly (ethylene oxide)/poly (ethylene oxide)/poly (ethylene oxide) (PEO-PPO-PEO) triblock copolymers, of which aqueous solutions undergo sol-gel transition at temperature above critical gel temperature (CGT), which has been extensively studied in tissue engineering [13,14,15]. To modulate the characteristics of Pluronic F127, synthetic modification has been reported by the incorporation of chitosan for the formulation of nanoparticles [16,17,18], drug release [19,20] and the development of injectable biomaterials [21,22,23]. On the other hand, another effective modification that has been carried out on different hydrogels to improve the mechanical characteristics and functionality corresponds to the use of various components co-polymerized or immersed in hydrogels, where the use of liposomes is emphasized, which provide a system of controlled release of active substances that promote the growth and development of the transplanted cells [24].

The design of hydrogels for use in neural tissue engineering must consider the sensitive relationship between the neural cells and their extracellular matrix, with which physical properties associated with the rigidity and interconnectivity of the three-dimensional network, together with surface parameters such as hydrophobicity, surface energy and topology, are critical for the correct performance of transplanted cells. Therefore, it is critical to know the effects of the combination of components on the physical parameters and how these affect the development of cells in vitro [25,26,27,28,29,30,31].

For the study of neural cell responses to different materials, one of the most used models corresponds to the use of the PC12 cell line, which derives from a rat adrenal pheochromocytoma, which has an embryonic origin of the neural crest and has a mixture of neuroblasts and eosinophilic cells [32]. These cells respond to neural growth factor (NGF), which produces phenotypic changes with characteristic properties of sympathetic neurons, including the promotion of neurite outgrowth [33].

In this research, we evaluated the capabilities of the Pluronic F127/Chitosan hydrogel formulations together with the incorporation of liposomes to induce cell development, which could represent a new strategy for its use in neural tissue engineering. Our research includes the determination of physical effects related to the rheological characteristics of hydrogels and its relation to the proliferation and development of neurites of the PC12 cell line.

## 2. Results and Discussion

### 2.1. Rheological Analysis and DSC Measurements

#### 2.1.1. Effect of Chitosan on Pluronic F127

**Figure 1:**
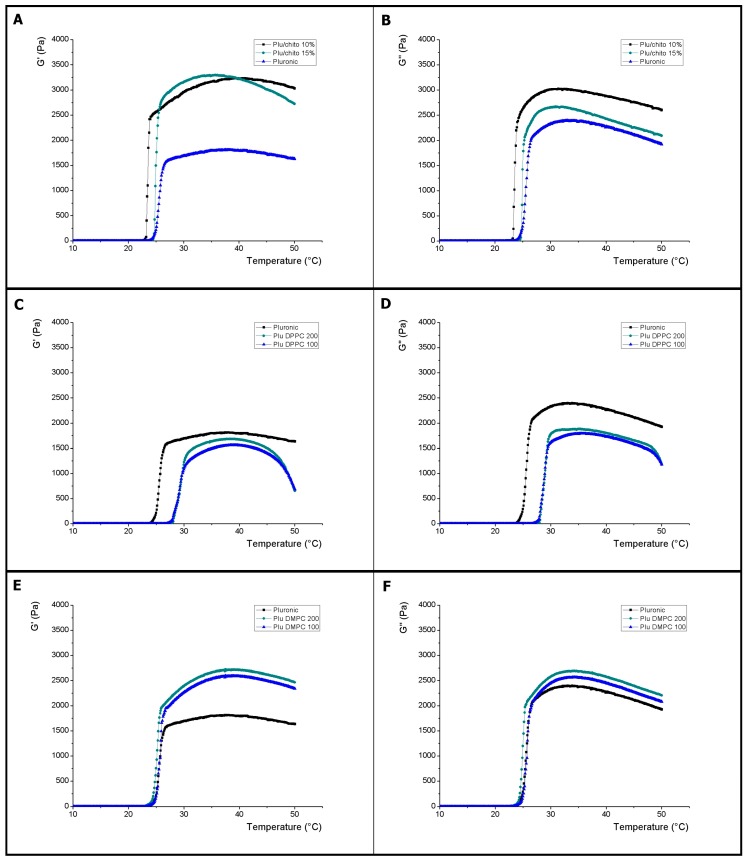
Variation of elastic (*G*′) and viscous (*G*″) modules as a function of temperature. (**A**,**B**) corresponds to the Pluronic F127 control and the effect of incorporation of chitosan (F10% and F15%); (**C**,**D**) correspond to the effect of incorporation of DPPC-composed Liposomes made by extrusion with 100 and 200 nm membranes (plu 100 and plu 200, respectively) on Pluronic F127; (**E**,**F**) correspond to the effect of incorporation of DMPC composed Liposomes made by extrusion with 100 and 200 nm membranes (plu 100 and plu 200, respectively) on Pluronic F127.

The Figure 1 shows the characteristic evolution of the elastic (*G*′) and viscous (*G*″) modules as a function of the temperature increase for control, F10% and F15% samples (Pluronic F127 with 10% and 15% of the F127-Chitosan complex respectively). The increase of the values of *G*′ and *G*″ modules for the control (Pluronic F127 20%) was observed at an average temperature of 24.43 °C, similar to that reported for this analysis [34]. For samples F15%, the *G*′ and *G*″ rise temperature (24.61 °C) similar to the control, however, the magnitude of *G*′ doubles to the control by averaging 3.280 (Pa) at its peak and *G*″ is significantly higher. For samples F10%, the *G*′ increase process begins at a temperature of 23.1 °C and the magnitude of *G*′ and *G*″ at its peak are significantly greater than the control but similar to F15%. See Table 1. The results agree with the increase in the viscoelastic properties observed with the formation of the Pluronic F127-chitosan complex [35].

The complexity of the rheological behavior of triblock copolymers (PEO-PPO-PEO) is due to sol-gel phase change characteristics, where the critical point of the process is the micellation prior to viscoelastic change [36,37]. The formation of micelles can be studied by DSC in determining the points where the endothermic change occurs, which corresponds to the reorganization of the monomers, due to hydrophobic interactions, where the PPO nucleus is arranged surrounded by a PEO crown as a result of the dehydration and aggregation of unimers [22,37,38]. Table 2 shows the characteristic T_onset_ and T_end_ determinations for the micellation process for each of the samples studied [39]. For the samples studied, the micellation process is consistent with the rheological measurements and the estimation of the gelation temperature (*G*′ = *G*″, Table 1). The insertion of the Pluronic F127-Chitosan complex mixed with Pluronic F127 forms a stable solution with particular physical properties reported as the formation of stable nanometric aggregates derived from the formation of new intermolecular bonds [19]. For the formulations used in this study mixtures of Pluronic F127/Pluronic F127-Chitosan with a final concentration of 20% resulted in a viscoelastic modification of the hydrogels probably derived from the interaction of Pluronic F127 with Pluronic F127-Chitosan complex by complementation of the NH groups of the chitosan and the bond between the oxygen atoms of Pluronic F127 ether groups and the protons of the water, which has been reported as a molecular mechanism associated with the physical properties of the Pluronic F127-Chitosan complex [40,41], as well as in the direct mixture of Chitosan with Pluronic F127 [20].

#### 2.1.2. Effect of Size and Composition of Liposomes on Pluronic F127

In this research, the effects of liposome incorporation on the thermal characteristics of Pluronic F127 were studied using two types of lipids (DMPC and DPPC) and two extrusion sizes in the manufacture of liposomes (100 nm and 200 nm). Both DMPC and DPPC correspond to phosphatidylcholine phosphates, saturated, carbon chain of 14 and 16 respectively. Liposome sizes and polydispersity (pd) were measured using DLS. In the DMPC liposome formulations the size results were on average 119 nm (pd = 0.12) and 224 nm (pd = 0.19) for the use of 100 nm and 200 nm pore size membranes respectively, while that for the DPPC-composed liposomes the sizes were 136 nm (pd = 0.22) and 247 nm (pd = 0.29) for the use of 100 nm and 200 nm pore size membranes respectively, indicating a homogeneous distribution of sizes and a small difference in size between the different lipids used.

The Figure 1 shows the thermal evolution of the elastic (*G*′) and viscous (*G*″) modules for formulations with liposomes. For the samples composed of DMPC there is no significant variation in the onset temperature of the viscoelastic phenomena with respect to the control, however, both the *G*′ and *G*″ modulus were significantly higher at the control, where the size of the liposomes did not significantly influenced the results (see Table 1). The T_onset_ and T_end_ determinations are correlated to a behavior similar to that shown in the mixtures with chitosan, in which there is a relation with an increase of the micellation time before the gelation process, being estimated in a coherent way the temperature of transition sol-gel (See Table 1 and Table 2).

On the other hand, the liposomes composed by DPPC caused a particular behavior in the hydrogels where there is a displacement toward a higher temperature close to 29 °C, for the beginning of the viscoelastic process together with an increase in the estimation of the temperature of change of Sol-gel phase (See Table 1). The determination of T_onset_ and T_end_ for the samples with liposomes composed by DPPC on Pluronic F127 show the extensive micellation process prior to the beginning of viscoelastic processes (see Table 2).

The results obtained present evidence of a relationship between the characteristics of the lipids used and the thermal properties of Pluronic F127. In this sense, the degree of saturation and the hydrophobicity of the apolar lipid tails seems to exert an effect on the micellation process and the hydrogel structure. In liposome membranes, the length of the aliphatic chains and the degree of saturation of the lipids is related to the degree of hydrophobicity that leads to greater stiffness, lower permeability of the membranes, lower fluidity and higher transition temperature (Tc) between the crystalline and fluid membranes states [42,43]. In the unsaturated chains, due to the increased the interaction area, the interactions between the chains are larger, which could provide a structural order that where reaches a micellation point at a higher temperature, which would explain the behavior of the samples composed by Pluronic F127 and liposomes composed by DPPC (See Table 1 and Table 2) [44,45]. These results may explain the differences between the data obtained using soybean lipids, under similar experimental conditions the thermal effects on Pluronic F127 are different from those presented in this investigation due to the use of polyunsaturated lipids [46]. The reduction of the *G*′ and *G*″ modules of the Pluronic F127 samples with DPPC-composed liposomes would be related to the liposome characteristics as a function of the stiffness of their membranes, which would imply less interaction in the hydrogel network, has already been reported for other nanoparticles [47].

#### 2.1.3. Effect of Size and Composition of Liposomes on Pluronic F127/Pluronic F127-Chitosan

**Figure 2:**
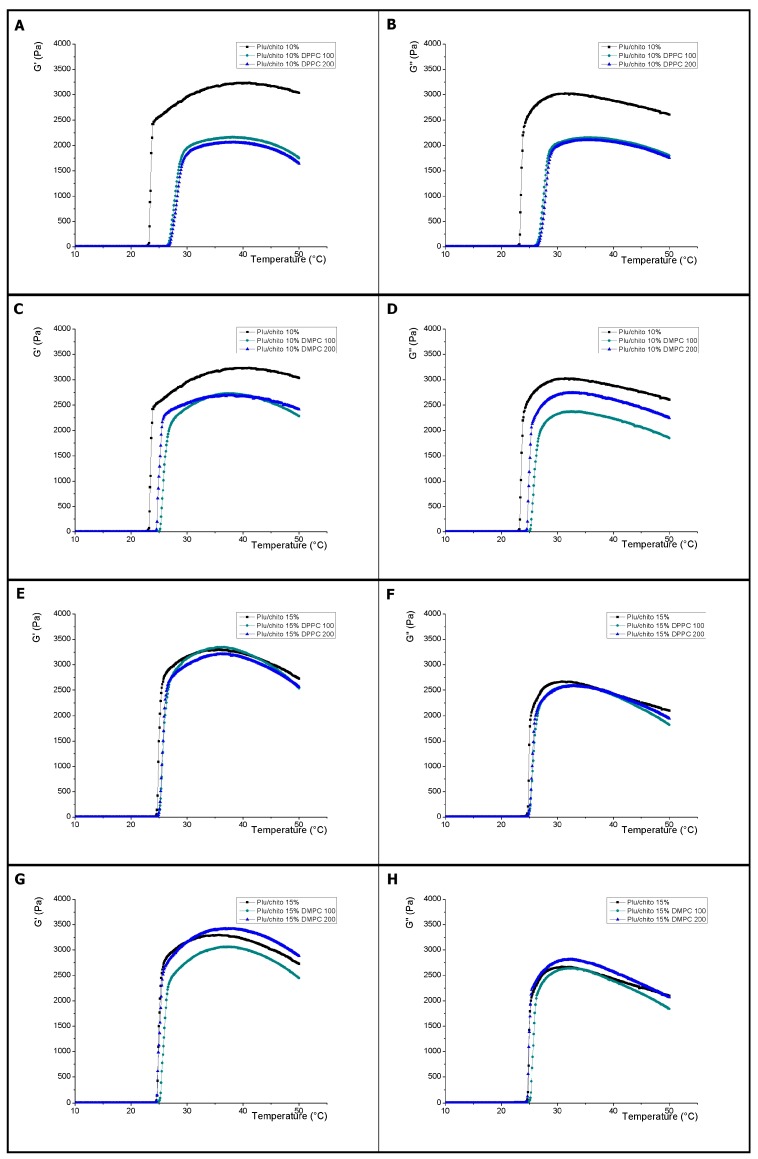
Variation of elastic (*G*′) and viscous (*G*″) modules as a function of temperature. (**A**,**B**) correspond to the effect of incorporation of DPPC composed liposomes built by extrusion with membranes of 100 and 200 nm (pluronic 10% DPPC 100 and pluronic 10% DPPC 200, respectively) on Pluronic F127/Pluronic F127-Chitosan 10% hydrogels; (**C**,**D**) correspond to the effect of incorporation of DMPC composed liposomes built by extrusion with membranes of 100 and 200 nm (pluronic 10% DMPC 100 and pluronic 10% DMPC 200, respectively) on Pluronic F127/Pluronic F127-Chitosan 15% hydrogels; (**E**,**F**) correspond to the effect of incorporation of DPPC composed liposomes built by extrusion with membranes of 100 and 200 nm (pluronic 15% DPPC 100 and pluronic 15% DPPC 200, respectively) on Pluronic F127/Pluronic F127-Chitosan 15% hydrogels; (**G**,**H**) correspond to the effect of incorporation of DMPC composed liposomes built by extrusion with membranes of 100 and 200 nm (pluronic 15% DMPC 100 and pluronic 15% DMPC 200, respectively) on Pluronic F127/Pluronic F127-Chitosan 15% hydrogels.

In this research the rheological and thermal behavior characteristics of the F10% and F15% samples were evaluated in the presence of liposomes. Figure 2 shows the effect of liposomes on viscoelastic parameters of F10%. Inclusion of liposomes composed of DMPC and DPPC significantly reduced the values of the *G*′ and *G*″ modules compared to the control. Also shown is a relationship between liposome sizes for *G*″ values when liposomes were conformed with DMPC, which implies redistribution of the hydrogel network. Regarding the magnitude of the *G*′ and *G*″ modules, the samples containing both DPPC and DMPC liposomes do not show a significantly different thermal behavior than shown in blends with Pluronic F127 which indicates that there is no significant effect mediated by Inclusion of chitosan, however, there is a shift towards a higher temperature for the onset of the viscoelastic phenomenon which is significantly greater with the samples containing liposomes composed by DPPC, which is evidenced by the determination of the gelation point (See Table 1). As with samples containing only Pluronic F127, the determination of T_onset_ and T_end_ shows the beginning of the broad micellation process prior to the commencement of the viscoelastic change for liposomes composed of DMPC and DPPC (see Table 2).

In Figure 2, the effect on the *G*′ and *G*″ modules of liposomes into F15% is shown. For both samples with liposomes composed of DPPC and DMPC, the results show no significant difference against the F15% control. For samples composed of DMPC liposomes, there is a significant difference of the *G*′ modulus for the extrusion sizes 100 nm and 200 nm, however there are no significant differences in the beginning of the viscoelastic change processes with respect to the F15% control, which implies A predominance of the thermal processes regulated by the concentration of the Pluronic F127-chitosan complex (see Table 1), which is also evident in the study of micellation processes (see Table 2). Several investigations have studied the structural relationships that determine the changes in the physical properties of Pluronic F127, where molecular weight and hydrophobicity conditions can modify the intermolecular micellar distances [48]. Our results indicate that the inclusion of liposomes in the hydrogel structure produces significant changes in the thermal properties of the hydrogel dependent on the concentration of Chitosan.

### 2.2. In Vitro Studies

Due to its high permeability, solubility and network morphology, Pluronic F-127 is considered a scaffold with characteristics suitable for the delivery and maintenance of cells [14,49]. However, not all cell types respond in the same way to three-dimensional culture [50], which also has been demonstrated with PC12 cells [51]. Different studies show that neuronal regeneration depends on the three-dimensional environment in a manner equivalent to biochemical signals soluble in two-dimensional cultures [52]. In this research we have studied how the addition of Chitosan and liposomes into Pluronic F127 affect the structural characteristics of the hydrogel and how these differences affect the behavior of PC12 cells.

**Figure 3:**
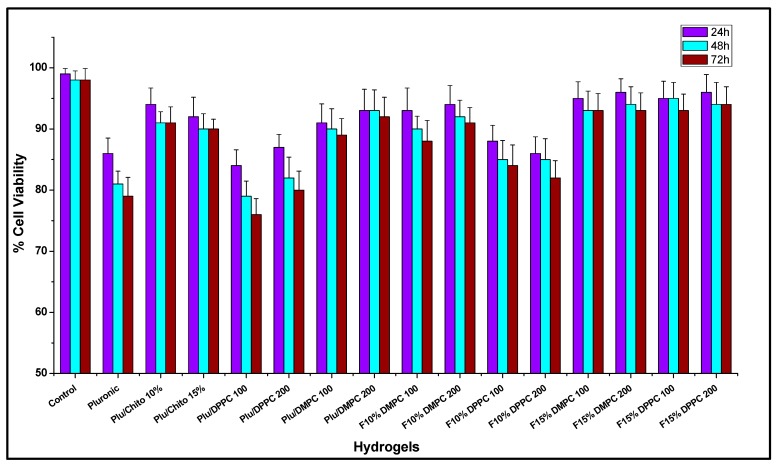
Cell viability of PC12 cells encapsulated in different hydrogels for 24 to 72 h.

**Figure 4:**
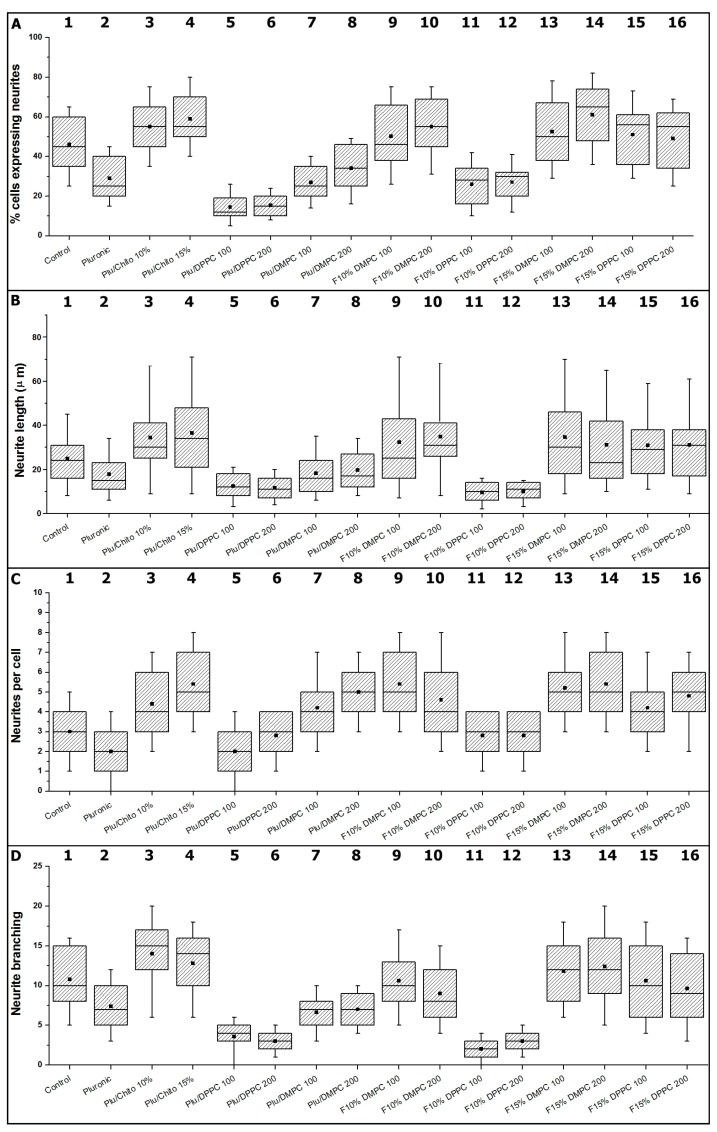
Analysis of neurite outgrowth parameters; Box-and-whisker plots of (**A**) Per cent cells expressing neurite; (**B**) Neurite length; (**C**) Neurites per cell; (**D**) Neurite branching. (n = 5, *p* < 0.005). Boxes enclose 25th and 75th percentiles of each distribution and are bisected by the median; whiskers indicate 5th and 95th percentiles. Boxes from 1 to 4 represent of effect of chitosan on pluronic F127 on PC12 cellular differentiation, boxes from 5 to 8 represent of effect of Liposomes on pluronic F127 on PC12 cellular differentiation, boxes from 9 to 12 represent of effect of Liposomes on Pluronic F127/Pluronic F127-Chitosan 10% on PC12 cellular differentiation and boxes from 13 to 16 represent of effect of Liposomes on Pluronic F127/Pluronic F127-Chitosan 15% on PC12 cellular differentiation.

For hydrogels composed exclusively of Pluronic F127, the results of cellular viability assessment and neural differentiation parameters show a significant reduction over the control sample (Figure 3 and Figure 4, boxes 1 to 4). The lower mechanical resistance, expressed through rheological studies and the low affinity of the hydrogel for NGF, make Pluronic F127 alone not suitable for delivery and release of neural cells [53], the incorporation of chitosan into Pluronic F127 (F15% and F10%) modifies the mechanical properties of the hydrogel, resulting in hydrogels with appropriate capacities for the maintenance and differentiation of PC12 cells [54,55]. The incorporation of new components to Pluronic F127 has been a strategy used in different investigations to improve the mechanical properties and alter the topology of the hydrogel [56,57,58] and improve the capabilities of using PC12 cells [59].

**Figure 5:**
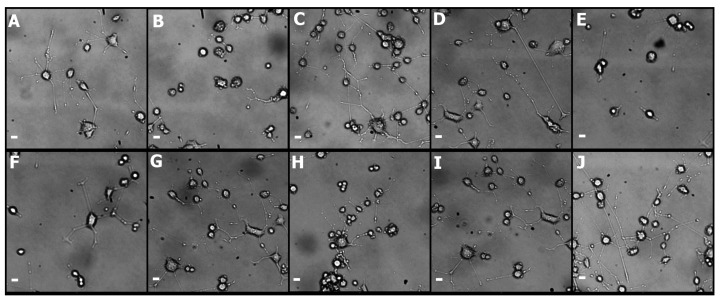
Differentiation of PC-12 cells in the presence of NGF. Photomicrographs representative of different hydrogels. (**A**) Differentiation control medium; (**B**) Pluronic F127; (**C**) Pluronic F127/Pluronic F127-Chitosan 10%; (**D**) Pluronic F127/Pluronic F127-Chitosan 15%; (**E**) Pluronic F127 DPPC liposomes; (**F**) Pluronic F127 DMPC liposomes; (**G**) Pluronic F127/Pluronic F127-Chitosan 10% DMPC liposomes; (**H**) Pluronic F127/Pluronic F127-Chitosan 10% DPPC liposomes; (**I**) Pluronic F127/Pluronic F127-Chitosan 15% DPPC liposomes; (**J**) Pluronic F127/Pluronic F127-Chitosan 15% DMPC liposomes. The bar in the photomicrographs corresponds to 20 μm.

On the other hand, the parameters related to neurogenesis show that the incorporation of chitosan produces hydrogels with characteristics appropriate for the neural differentiation (see Figure 4). Neurite outgrowth in PC12 Cells, depends on biochemical [60] and physical factors, the latter associated with the topology and nanomechanics of three-dimensional matrices, which affects the anisotropic level of surfaces in contact with cells [61,62]. Although there is greater knowledge of mechanotransduction processes for various tissues [63], for soft tissue, such as neural tissue, there is no consensus on the material’s hardness and neurite outgrowth processes [64,65,66,67], however, it is possible to consider, according to which stiffness of the hydrogels with chitosan studied here are in ranges appropriate for the neurogenesis of PC12 cells (see Figure 5) [68].

The incorporation of liposomes into the Pluronic, F10% and F15% formulations generates different effects on the viability of PC12 cells, where there is a significant reduction in lipid-type-dependent viability and independent of size for Pluronic and F10% formulations, where the reduction in viability was significant with the use of DPPC liposomes. For the F15% formulations, there is no significant difference between the use of different liposomes (Figure 3). Thus, it is possible to determine that the concentration of the Pluronic F127-Chitosan complex in the hydrogels is determinant to the capacities of the formulations in order not to significantly alter the viability of PC12 cells, an effect similar to those discussed previously in this manuscript. The use of liposomes immersed in hydrogels as a means for cell delivery in tissue engineering is a strategy that has great importance for various therapeutic approaches [69], however, these liposomes can alter the mechanical characteristics of hydrogels, which is highly sensitive for some cell types [70,71].

The incorporation of liposomes on Pluronic F127, where there is a significant reduction of hydrogel capacities to promote neural development (Figure 4, boxes 5 to 8). The incorporation of liposomes on the F10% hydrogel, where the parameters show significant differences between the different types of liposomes, independent of size, where liposomes composed by DPPC significantly induce the reduction of hydrogel capacities to favor the neurogenesis processes of PC12 cells (Figure 4, boxes 9 to 12). In this respect, hydrogels containing DMPC do not modify the initial properties of the hydrogel to favor the of neurite outgrowth processes, both results consistent with the physical parameters observed for both hydrogels.

For the F15% hydrogels containing DMPC and DPPC liposomes (Figure 4, boxes 13 to 16). The observed results show that incorporation of liposomes does not significantly affect the initial properties of the hydrogel to favor neurite outgrowth in PC12 cells. The results obtained with the incorporation of liposomes in the different hydrogels demonstrate the complexity of possible interactions within the polymer matrix, depending on each of the elements that make up the formulation, which is evidenced through contradictory results in the literature [72,73,74]. Figure 5 summarizes the neurogenesis processes on PC12 cells for the different hydrogel configurations. The lengths of neurites, the number of neurites, the branches and the presence of neurites per cell are observable.

## 3. Materials and Methods

### 3.1. Materials

Chitosan, 1-Ethyl-3-(3-dimethylaminopropyl)-carbodiimide (EDC), N-hydroxysuccinimide (NHS), succinic anhydride, Pluronic^®^ F127 and 4-dimethylaminopyridine (DMAP) were purchased from Aldrich (Sigma Aldrich, St. Louis, MO, USA). 1,2-dimyristoyl-sn-glycero-3-phosphocholine (DMPC) and 1,2-dipalmitoyl-sn-glycero-3-phosphocholine (DPPC) were purchased from Avanti Polar Lipids (Alabaster, AL, USA). All other reagent grade chemicals and solvents were used as received and were obtained from commercial sources.

### 3.2. Synthesis of Pluronic F127-Chitosan

**Figure 6:**
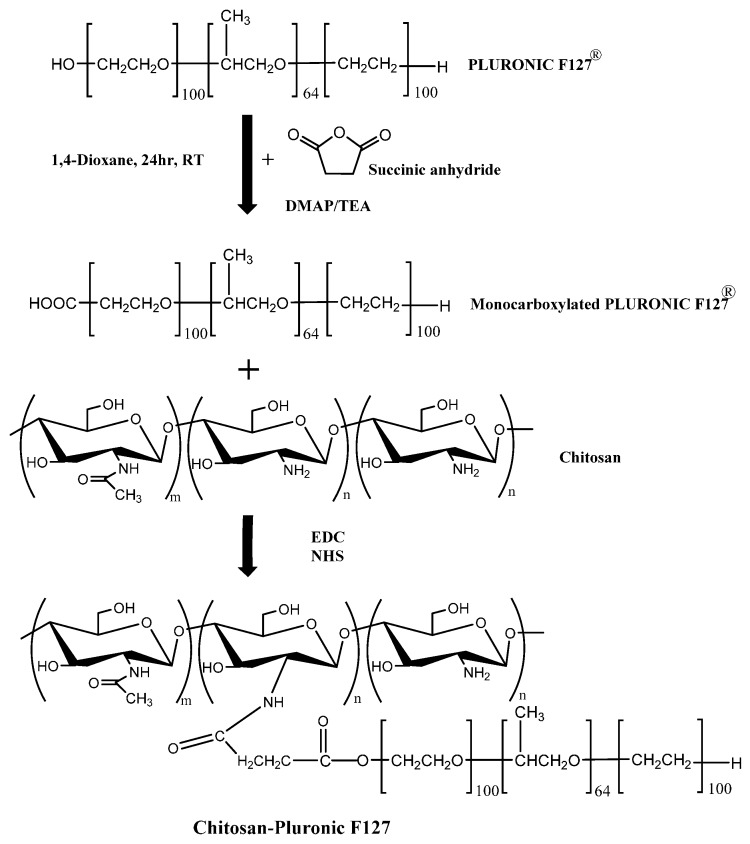
Synthetic route of Pluronic F127-Chitosan using EDC/NHS.

Pluronic F127 was carboxylated with succinic anhydride to produce monocarboxy Pluronic (MP) in the presence of DMAP and TEA. MP was coupled with chitosan by EDC/NHS at R.T. For 24 h. The product of the synthesis was dialyzed against water for 3 days and finally lyophilized (see Figure 6). The lyophilized product was mixed with Pluronic F127 at 4 °C for 24 h at a concentration of 10% and 15% to form the F10% and F15% hydrogels. The final concentration of Pluronic F127 control and all formulations was 20% [21,75].

### 3.3. Liposome Preparation

Liposomes were formed by lipid (DMPC and DPPC) solubilization with a mixture of chloroform and methanol (1:1, v/v), dried under a stream of nitrogen in a conical tube, and kept under high vacuum for 12 h to remove traces of organic solvent. The suspension (10 mg/mL) was subjected to three freeze/thaw cycles to promote the formation of larger lipid aggregates. The lipid aggregates were then extruded at 40 °C through a polycarbonate membrane, with an etched pore size of 100 nm and 200 nm, using the Avanti Mini-Extruder apparatus (Avanti Polar Lipids) The final concentration of liposomes in the different formulations was 1 mg/mL. The size distribution of the liposomes was determined by a dynamic light scattering analysis (DLS) using a Zetasizer NanoZS (Malvern Instruments, Malvern, UK) in triplicate at 25 °C for 60 s, with an average count rate of 280 kcps [76,77].

### 3.4. Rheological Analysis of Thermosensitive Hydrogels

For rheological measurements of the hydrogels, the elastic (*G*′) and viscous (*G*″) shear modulous were monitoring relating to changes in temperature at frequency of 0.1 Hz using Discovery Hybrid Rheometer HR2 (TA Instrument, Crawley, UK). The hydrogels solutions were maintained for 10 min at 10 °C before each triplicate analysis. For the oscillatory shear rheological measurement, parallel plate geometry was used (plate diameter = 30 mm, gap = 150 μm) The samples were placed in the plate of rheometer using temperature of 1 °C per minute over the range 10–50 °C [78].

The rheological analysis of oscillatory deformation is performed as a function of temperature, which allows the measurement of the complex shear modulus (G*), which estimates the fundamental characteristics of viscoelasticity of the materials, Through the use of the equation established by its real and imaginary components as:

(1) $$G^{*}\left(t\right)=G^{\prime }\left(t\right)+iG^{\prime \prime }\left(t\right)$$

where *G*′ corresponds to the elastic modulus, which represents the reversibly stored deformation energy and *G*″ viscous modulus, represents a measure of irreversibly dissipated energy. On the other hand, the degree of viscoelasticity of the samples is measured by the dimensionless parameter *tanδ* as:

(2) $$tan\delta =\frac{G^{\prime \prime }}{G^{\prime }}$$

The estimation of *tanδ* allows to identify the relation between the viscous and elastic characteristics of the materials, allowing to establish how the molecular interactions of the components affect, mainly, the degree of crosslinking of the material [78]. The relationship between the viscous and elastic components allows to establish the gelling temperature when *G*′ = *G*″ [34].

### 3.5. Measurements of Differential Scanning Calorimetry (DSC)

DSC experiments were performed with a differential scanning calorimeter (Mettler Toledo, Zurich, Switzerland). Hydrogel samples, of 20 mg in weight were hermetically sealed in aluminum pann of 40 μL. The samples were stabilized for 5 min at 10 °C prior to thermal scanning, and subsequently heated at 1 °C/min from 10 °C to 50 °C. The DSC was previously calibrated using indium as standard and an empty pan was used as reference under N2. The micellization process was determined on the onset and end of the endothermic transition observed on the heating scan per triplicate. The estimation was performed using the MODDE software (Umetrics, Umeå, Sweden) [39].

The micellation process is studied by DSC under conditions equivalent to rheological measurements. In the conformation of thermograms, an endothermic phenomenon is observed prior to the change of the viscoelastic characteristics of the hydrogels, where the temperature is determined where the phenomenon starts as T_onset_ equivalent to critical micelle temperature (CMT) and T_end_ as the temperature where the formation of micelles ends and that limits the endothermic area.

### 3.6. Culture of PC12 Cells

PC12 cells, a rat adrenal pheochromocytoma cell line that is induced by neurite growth factor (NGF) into a neuronal phenotype cells were designated as received at passage 1 and used at passage 6–7. PC12 cells were maintained tissue culture-treated polystyrene plates coated with type I collagen (Thermo Fisher Scientific, Waltham, MA, USA) and RPMI 1640 Medium (ATCC modification) medium with 2 mM l-glutamine and 1.5 g L^−1^ sodium bicarbonate (Thermo Fisher Scientific, USA), which was supplemented with 15% horse serum and 5% fetal bovine serum (Sigma Aldrich, USA), 100 units/mL penicillin and 100 μg mL^−1^ streptomycin (Gibco, Waltham, MA, USA). To subculture, the cells were removed in trypsin-EDTA (Gibco, Waltham, MA, USA). The medium was replaced every 3 days.

### 3.7. Analysis of Viability of PC12 Cells and Neurite Outgrowth on Hydrogels

The viability assays were performed using the WST-1 colorimetric assay, 90 μL medium (45,000 cells per well) and 10 μL WST-1 reagent were seeded in 96-well plates. Measurements were optimized by incubation time to 2 h. To determine viability, the cells were added on the hydrogels in a 1:9 ratio, incubated for 24, 48 and 72 h. Samples were analyzed in triplicate.

Neurite growth analysis was performed in 6-well plates, sterile hydrogels were deposited at 37 °C with PC12 cells encapsulated (40,000 cells) supplemented with NGF-supplemented medium (50 ng/mL) and was allowed to bind for 10 h at 37 °C and the medium was withdrawn, incubated for 48 h, 3% glutaraldehyde was added in PBS, the cells were then rinsed and stored in fresh at 4 °C until analysis microscopic. For the control, a treatment with collagen tipe I was used on the surfaces of each well. The images were captured under phase contrast microscopy at 10 random locations. The length of neurite, the branching, the number of neurites per cell and percent of cells expressing neurites were analyzed with ImageJ software. Criteria were used for the image analysis provided by the software and those used in other studies [68].

### 3.8. Statistical Analysis

The anaisis of the results of the rheological tests, of calorimetry and cellular viability were performed as mean ± standard error of the mean. For tests of multiple comparisons (ANOVA) with a significance level of *p* < 0.05, (n = 3).

For the analysis of the parameters of neurocygenesis, scores are not normally distributed data are shown by box-and-whisker plot displaying the medians, 25% quartiles, 75% quartiles. Statistical significance was determined by performing Kruskal–Wallis test (n = 5).

## 4. Conclusions

The present research provides key information for the design of neural cell delivery systems in tissue therapy. The results indicate the dependence of hydrogel stiffness and the ability to promote differentiation of PC12 cells depending on the type of liposome, the presence of the chitosan-Pluronic F127 complex and the independence of the size of embedded liposomes. To date no formulations have been reported that include the Pluronic F127-Chitosan complex mixed with Pluronic F127 as a useful material in the possible administration of neural cells, which, this research could represent the beginning of a scan line with results that can Establish a new area of material development.

## Figures and Tables

**Table 1:** Characterization of viscoelastic parameters of hydrogels.

Sample	*G*′ = *G*″ (°C)	SD (±)	*G*′ 37 °C (Pa)	SD (±)	*G*″ 37 °C (Pa)	SD (±)	*tanδ* 37 °C	SD (±)
Pluronic F127 control	25.62	0.37	1798	103	2346	28	1.299	0.272
PLU/CHITO 10%	23.89	0.29	3189	106	2944	28	0.917	0.264
PLU/CHITO 15%	24.57	0.33	3297	143	2536	35	0.772	0.245
PLU DPPC 100	28.41	0.26	1545	121	1781	27	1.153	0.223
PLU DPPC 200	28.38	0.31	1674	106	1859	31	1.111	0.292
PLU DMPC 100	24.73	0.52	2526	133	2526	34	0.979	0.256
PLU DMPC 200	24.62	0.43	2651	145	2561	42	0.981	0.29
PLU/CHITO 10% DPPC 100	28.57	0.49	2156	128	2144	36	0.995	0.281
PLU/CHITO 10% DPPC 200	28.71	0.63	2059	143	2097	41	1.021	0.287
PLU/CHITO 10% DMPC 100	26.89	0.38	2725	166	2303	37	0.844	0.223
PLU/CHITO 10% DMPC 200	26.68	0.42	2690	139	2677	38	0.995	0.273
PLU/CHITO 15% DPPC 100	25.04	0.25	3339	146	2517	30	0.754	0.205
PLU/CHITO 15% DPPC 200	25.13	0.27	3206	172	2514	42	0.784	0.244
PLU/CHITO 15% DMPC 100	25.36	0.23	3061	137	2518	39	0.822	0.285
PLU/CHITO 15% DMPC 200	24.56	0.19	3.421	158	2.689	47	0.787	0.297

**Table 2:** Micellation process. Transition temperatures for the different hydrogels studied.

Sample	T_onset_ (°C)	SD (±)	T_end_ (°C)	SD (±)
Plu control	11.47	0.61	24.31	0.47
PLU/CHITO 10%	8.61	0.21	23.17	0.26
PLU/CHITO 15%	8.07	0.19	24.37	0.25
PLU DPPC 100	20.12	0.31	28.01	0.26
PLU DPPC 200	20.43	0.24	27.92	0.23
PLU DMPC 100	16.23	0.34	25.23	0.42
PLU DMPC 200	16.31	0.37	24.76	0.35
PLU/CHITO 10% DPPC 100	12.37	0.42	20.71	0.26
PLU/CHITO 10% DPPC 200	12.81	0.37	21.49	0.34
PLU/CHITO 10% DMPC 100	11.12	0.43	20.93	0.32
PLU/CHITO 10% DMPC 200	11.93	0.33	20.64	0.29
PLU/CHITO 15% DPPC 100	9.79	0.17	25.19	0.28
PLU/CHITO 15% DPPC 200	12.68	0.31	20.92	0.32
PLU/CHITO 15% DMPC 100	15.09	0.48	21.03	0.35
PLU/CHITO 15% DMPC 200	12.54	0.35	20.56	0.38
